# Supplementary material for: Dimerization of the β-Hairpin Membrane-Active Cationic Antimicrobial Peptide Capitellacin from Marine Polychaeta: An NMR Structural and Thermodynamic Study
Source: Biomolecules. 2024 Mar 11;14(3):332. doi: 10.3390/biom14030332 (PMC10968102; doi:10.3390/biom14030332)
Supplement: Supplementary file 1 [file biomolecules-14-00332-s001.zip › biomolecules-2854097-supplementary.pdf]

Supplementary materials

Dimerization of the β-Hairpin Membrane-Active Cationic Antimicrobial Peptide Capitellacin from Marine Polychaeta: An NMR Structural and Thermodynamic Study

Pavel A. Mironov 1,2, Alexander S. Paramonov 1, Olesya V. Reznikova 1, Victoria N. Safronova 1, Pavel V. Panteleev 1,3, Ilia A. Bolosov 1, Tatiana V. Ovchinnikova 1,3 and Zakhar O. Shenkarev 1,3,4,*

1 M.M. Shemyakin & Yu. A. Ovchinnikov Institute of Bioorganic Chemistry, Russian Academy of Sciences, 117997 Moscow, Russia; mironov@nmr.ru (P.A.M.); apar@nmr.ru (A.S.P.); olesya-r@list.ru (O.V.R.);
victoria.saf@ibch.ru (V.N.S.); p.v.panteleev@gmail.com (P.V.P.); bolosoff@gmail.com (I.A.B.); ovch@ibch.ru (T.V.O.)
2 Interdisciplinary Scientific and Educational School of Moscow University "Molecular Technologies of the Living Systems and Synthetic Biology", Faculty of Biology, Lomonosov Moscow State University,
119234 Moscow, Russia
3 Phystech School of Biological and Medical Physics, Moscow Institute of Physics and Technology (State University), 141701 Dolgoprudny, Russia
4 International Tomography Center, Siberian Branch of the Russian Academy of Sciences, 630090 Novosibirsk, Russia
* Correspondence: zakhar-shenkarev@yandex.ru

Supplementary figures

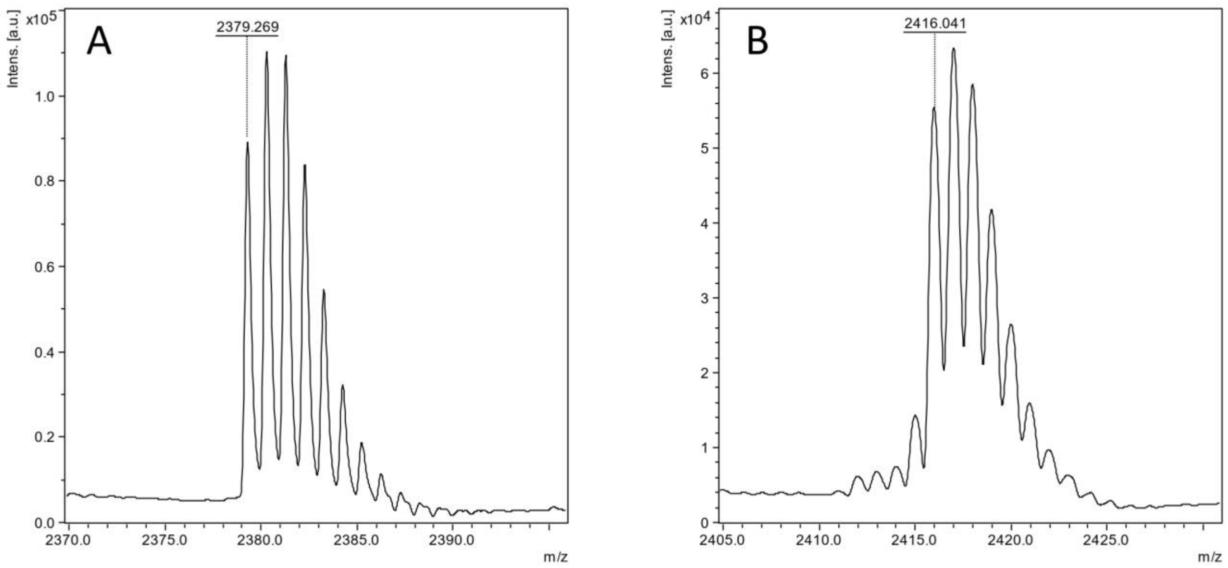

Figure S1. MALDI-MS analysis of the recombinant antimicrobial peptides. Molecular masses of ^{15}N-labeled capitellacin increased by 37 Da (A and B), indicating that all the ^{14}N atoms were substituted with stable isotopes ^{15}N. The experimentally measured *m*/*z* value of ^{15}N-labeled capitellacin matched well the $[M+H]^+$ value of its calculated molecular mass (2416.04 and 2416.16 Da, respectively).

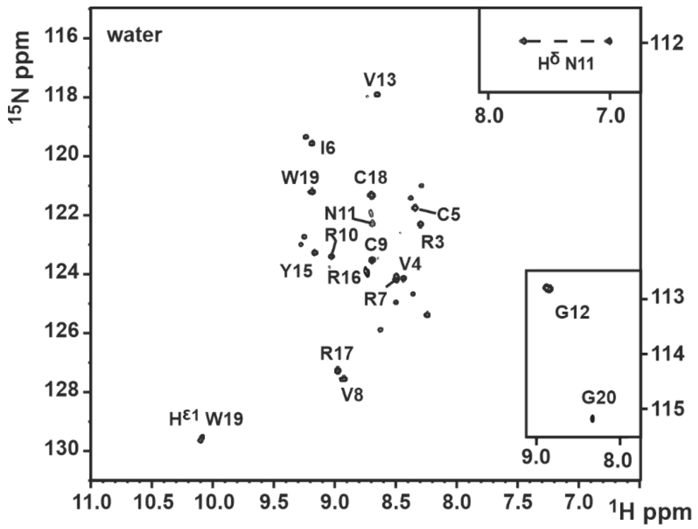

Figure S2. 2D sensitivity enhanced ^1H-^{15}N HSQC NMR spectrum of ^{15}N-labeled capitellacin in water (0.07 mM, pH 5.4, 45°C). Additional NMR signals (unassigned) belongs to the *cis*–Ser1–Pro2 isomer of the peptide.

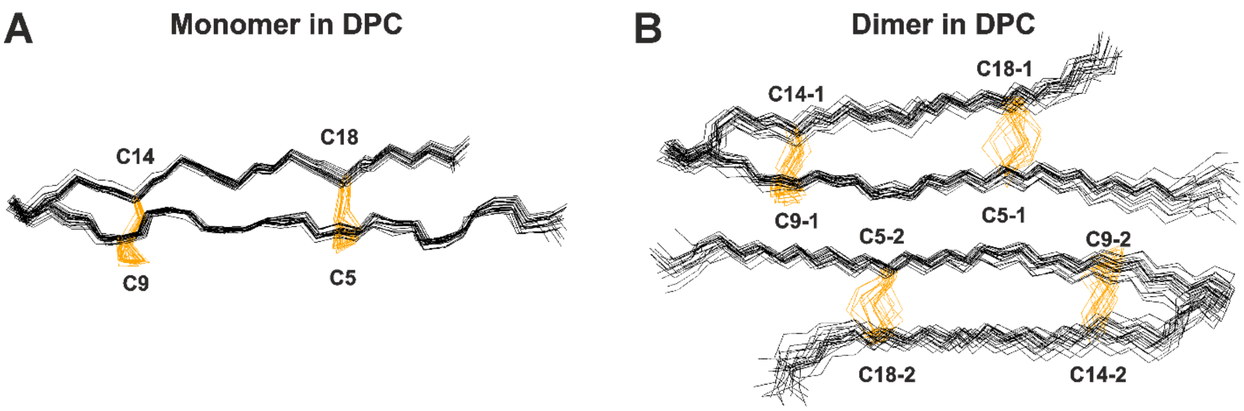

Figure S3. Sets of 20 structures calculated for the capitellacin monomer (**A**) and dimer (**B**) in DPC micelles (present work, PDB ID: 8B4R and 8B4S, respectively). Only backbone of the peptide is shown. Disulfide bonds are colored in orange.

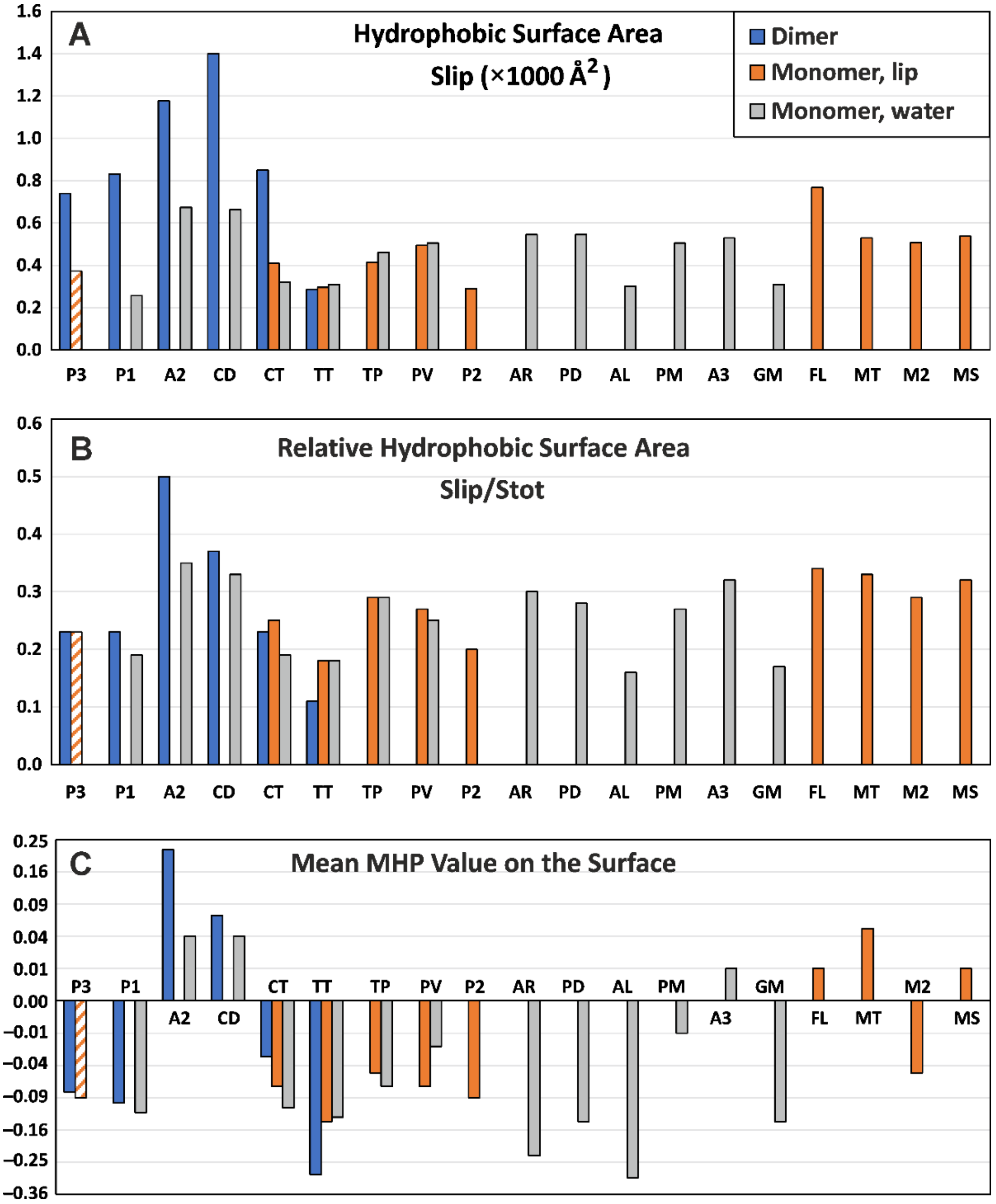

Figure S4. Some of the physicochemical properties of the β-hairpin and helical AMPs in monomeric form in water (*gray*), in monomeric form in membrane mimetic (*orange*) and in dimeric form in membrane mimetic (*blue*). **(A)** S_{lip}, surface area with strong hydrophobicity (where MHP > +0.2). **(B)** S_{lip}/S_{tot}, relative surface area occupied by hydrophobic regions. **(C)** Mean MHP value on the peptide surfaces. Please note the nonlinear vertical scale on the panel **(C)**. The Protegrin-3 monomer (orange hatched bars) was created by splitting NMR structure of Protegrin-3 dimer into two chains. Abbreviations: Protegrin-3 (P3), Protegrin-2 (P2), Protegrin-1 (P1), Arenicin-2 (A2), ChDode (CD), Capitellacin (CT), Thanatin (TT), Tachyplesin I (TP), Arenicin-1 [V8R] (AR), PcDode (PD), Alvinellacin (AL), Polyphemusin I (PM), PV5 (PV), Arenicin-3 (A3), Gomesin (GM), Fowlicidin-1 (FL), Melittin (MT), Magainin-2 (M2), MSI-594 (MS). Full list of the physicochemical properties and references to the peptide structures are shown in Tables S2A-D.

Supplementary tables

Table S1. Statistics for the best CYANA structures of capitellacin monomer and dimer forms in DPC micelles.

	monomer	dimer
**Distance and angle restraints**		
Total NOE contacts	131	194
Intraresidual	46	38
Sequential ($\|i – j\| = 1$)	45	86
Medium range ($1 < \|i – j\| < 5$)	15	24
Long range ($\|i – j\| > 5$)	25	36
Intermolecular	–	10
Hydrogen bond restraints (bonds/upper/lower)	8/16/16	20/40/40
S–S bond restraints (bonds/upper/lower)	2/6/6	4/12/12
Torsion angle restraints	21	46
Angle φ	14	32
Angle χ_1	7	14
Total restraints/per residue	196/9.8	344/8.6
**Statistics for calculated structures**		
Structures calculated/selected	20/200	20/200
CYANA target function (Å^2)	0.57± 0.07	0.29 ± 0.05
Violations of restraints		
Distance (> 0.2 Å)	2	0
Distance (> 0.4 Å)	0	0
Dihedral angles (> 1°)	0	2
r.m.s.d. (Å) overall		
Backbone	0.19 ± 0.10	0.28 ± 0.16
Heavy atoms	0.71 ± 0.11	1.07 ± 0.21

r.m.s.d. – root mean square deviation.

Table S2A. Physicochemical properties of the β-hairpin AMPs in monomeric form in water.

Peptide	Protegrin-1 a)	Protegrin-2	Protegrin-3 a)	Arenicin-2	Arenicin-1 [V8R]	Arenicin-3	ChDode b)	PcDode c)	Alvinellacin	Capitellacin	Thanatin	Tachyplesin I a)	Polyphemusin I a)	PV5 a)	Gomesin a,c)
Short name	**P1**	**P2**	**P3**	**A2**	**AR**	**A3**	**CD**	**PD**	**AL**	**CT**	**TT**	**TP**	**PM**	**PV**	**GM**
PDB ID, reference	1PG1, [29]	–	–	2JNI, [30]	5M9U, [21]	5V0Y, [31]	7ACE, [16]	7OSC, [16]	2LLR, [33]	7ALD, [23]	5XO4, [17]	1MA2, [18]	1RKK, [32]	1X7K, [19]	6MY2, [34]
Mw, Da	2155.7	1942.4	2056.5)	2772.3	2815.5	2613.1	3007.8	2778.5	2605.1	2379.8	2434.0	2263.7	2455.9	2610.2	2270.7
# a.a. res. / Cys	18/4	16/4	18/4	21/2	21/2	21/4	24/4	24/4	22/4	20/4	21/2	17/4	18/4	19/4	18/4
pI	10.66)	9.88	9.88)	10.85	11.30	9.25	10.41	10.45	10.16	10.41	10.47	9.93	10.33	10.83	9.93
GRAVY d)	–0.25	0.03	–0.02	–0.06	–0.49	–0.05	1.00	1.20	–0.46	–0.22	–0.90	–0.52	–0.83	–1.03	–1.06
Aromaticity	0.11	0.13	0.11	0.24	0.24	0.24	0.08	0.00	0.09	0.10	0.05	0.18	0.28	0.26	0.11
Charge at pH 7	+7	+6	+6	+6	+7	+4	+6	+4	+6	+5	+6	+7	+8	+9	+6
MHP e) (mean ± SD)	–0.12 ± 0.38			0.04 ± 0.34	–0.23 ± 0.60	0.01 ± 0.34	0.04 ± 0.38	–0.14 ± 0.50	–0.30 ± 0.48	–0.11 ± 0.36	–0.13 ± 0.33	–0.07 ± 0.40	–0.01 ± 0.31	–0.02 ± 0.32	–0.14 ± 0.32
MHP, Å^2 (sum)	–167.0			76.2	–418.9	16.9	75.5	–276.3	–562.6	–175.8	–226.7	–118.5	–22.3	–49.6	–247.4
S_{tot}, Å^2	1386			1903	1800	1646	2005	1940	1876	1644	1725	1600	1875	2025	1790
S_{lip}, Å^2 MHP > +0.2	257			673	545	529	663	545	302	320	309	462	505	505	310
S_{hyd}, Å^2 MHP < –0.2	611			507	845	470	561	771	1050	700	741	643	584	634	879
S_{lip} / S_{tot}	0.19			0.35	0.30	0.32	0.33	0.28	0.16	0.19	0.18	0.29	0.27	0.25	0.17

a) Calculated mass and charge take into account, and pI and GRAVY values do not take into account the *C*–terminal amidation.

b) Parameters are given for covalent dimer of ChDode.

c) Calculated mass and charge take into account, and pI and GRAVY values do not take into account interconversion of *N*–terminal Gln residue into cyclic pyroglutamate.

d) Average hydrophobicity index calculated according to Kyte and Doolittle hydrophobicity scale [25]. The maximum and minimum values of this index are +4.5 and –4.5 for poly-Ile and poly-Arg sequences, respectively.

e) Following values were calculated in the PLATINUM software [24] for the first structures from the NMR ensembles. MHP – the value of molecular hydrophobicity potential on the molecule surface. The maximal MHP values are approximately –2.0 (polar) and +2.0 (hydrophobic). S_{tot}, S_{lip}, and S_{hyd} – total, lipophilic (hydrophobic, where MHP > +0.2), and hydrophilic (MHP < –0.2) surface areas.

Table S2B. Surface hydrophobicity of the β-hairpin AMPs in monomeric form in DPC micelles.

	**Capitellacin**	**Thanatin**	**Tachyplesin I**	**PV5**	**Protegrin-2**	**Protegrin-3 chainA [f)]**
PDB ID, reference	8B4R	6AAB, [22]	1MA5, [18]	2B5K, [19]	2MUH, [20]	1ZY6, [13]
MHP (mean ± SD) [e)]	–0.07 ± 0.37	–0.14 ± 0.35	–0.05 ± 0.36	–0.07 ± 0.37	–0.09 ± 0.35	–0.09 ± 0.35
MHP (sum), Å^2	–118.1	–239.4	–76.6	–136.4	–129.2	–142.2
S_{tot}, Å^2	1665	1662	1413	1842	1429	1632
S_{lip} (MHP > +0.2), Å^2	409	296	415	494	290	373
S_{hyd} (MHP < –0.2), Å^2	632	735	547	802	499	713
S_{lip} / S_{tot}	0.25	0.18	0.29	0.27	0.20	0.23

f) Following values were calculated for the first chain from the first Protegrin–3 structure from the NMR ensemble.

Table S2C. Surface hydrophobicity of the β-hairpin AMPs in dimeric form in various membrane mimetics.

	**Protegrin-1**	**Protegrin-3**	**Arenicin-2**	**ChDode tetramer**	**Capitellacin**	**Thanatin**
Membrane mimetic	POPC	DPC	DPC	DPC	DPC	LPS
Type of dimer	Asymm. NC↑↑CN	Symm. NC↑↓CN	Asymm. CN↑↑NC	Symm. ↑↓↑↓	Symm. CN↑↓NC	Symm. CN↑↓NC
PDB ID, reference	1ZY6, [14]	2MZ6, [13]	2L8X, [15]	7ACB, [16]	8B4S	5XO9, [17]
MHP (mean ± SD) [e)]	–0.10 ± 0.37	–0.08 ± 0.35	0.22 ± 0.29	0.07 ± 0.40	–0.03 ± 0.38	–0.29 ± 0.41
MHP (sum), Å^2	–324.2	–252.6	743.6	252.9	–102.0	–746.9
S_{tot}, Å^2	3272	3095	3437	3841	3164	2590
S_{lip} (MHP > +0.2), Å^2	761	727	1702	1440	731	287
S_{hyd} (MHP < –0.2), Å^2	1454	1331	214	977	1063	1471
S_{lip} / S_{tot}	0.23	0.23	0.50	0.37	0.23	0.11

Table S2D. Physicochemical properties of the α-helical AMPs in monomeric form.

	**Fowlicidin-1**	**Melittin** [a)]	**Magainin-2** [a)]	**MSI-594**
Short name	**FL**	**MT**	**M2**	**MS**
Environment	Water/TFE 1:1	Crystal	DPC	LPS
PDB ID, reference	2AMN, [35]	2MLT, [36]	2MAG, [37]	2K98, [38]
Mw, Da	3141.9	2846.5	2465.9	2441.1
# a.a. res.	26	26	23	24
pI	11.60	12.02	10.00	10.70
GRAVY [d)]	–0.07	0.27	0.08	0.51
Aromaticity	0.12	0.04	0.13	0.04
Charge at pH 7	+8	+6	+4	+6
MHP (mean ± SD) [e)]	0.01 ± 0.40	0.05 ± 0.40	–0.05 ± 0.38	0.01 ± 0.44
MHP (sum)	30.4	86.2	–86.4	22.1
S_{tot}, Å^2	2272	1609	1749	1702
S_{lip} (MHP > +0.2), Å^2	768	529	507	537
S_{hyd} (MHP < –0.2), Å^2	691	464	664	594
S_{lip} / S_{tot}	0.34	0.33	0.29	0.32
